# Association of imaging‐defined brain age with disease severity and adverse outcomes in CADASIL

**DOI:** 10.1002/alz.71535

**Published:** 2026-06-02

**Authors:** Shao‐Lun Hsu, Pei‐Lin Lee, Kun‐Hsien Chou, Chen‐Yuan Kuo, Ching‐Po Lin, Yi‐Chu Liao, Chih‐Ping Chung, Yi‐Chung Lee

**Affiliations:** ^1^ Institute of Clinical Medicine National Yang Ming Chiao Tung University Taipei Taiwan; ^2^ Department of Neurology Fu Jen Catholic University Hospital New Taipei City Taiwan; ^3^ Institute of Neuroscience National Yang Ming Chiao Tung University Taipei Taiwan; ^4^ Brain Research Center National Yang Ming Chiao Tung University Taipei Taiwan; ^5^ Department of Education and Research Taipei City Hospital Taipei Taiwan; ^6^ Department of Neurology Taipei Veterans General Hospital Taipei Taiwan; ^7^ Department of Neurology National Yang Ming Chiao Tung University School of Medicine Taipei Taiwan; ^8^ Center for Intelligent Drug Systems and Smart Bio‐devices (IDS2B) National Yang Ming Chiao Tung University Hsinchu Taiwan

**Keywords:** brain age, brain age gap, cerebral autosomal dominant arteriopathy with subcortical infarcts and leukoencephalopathy (CADASIL), cerebral small vessel disease, *NOTCH3*

## Abstract

**INTRODUCTION:**

Cerebral autosomal dominant arteriopathy with subcortical infarcts and leukoencephalopathy (CADASIL) is caused by cysteine‐altering *NOTCH3* variants. We examined whether neuroimaging‐defined brain age is altered in CADASIL and its association with disease severity and outcomes.

**METHODS:**

A brain‐age prediction model was constructed using magnetic resonance imaging from 1482 healthy individuals and applied to 153 individuals with *NOTCH3* variants and 30 controls. Brain age gap (BAG) was calculated as predicted minus chronological age. Associations between BAG, imaging markers, and clinical outcomes were analyzed.

**RESULTS:**

Individuals with *NOTCH3* variants exhibited significantly higher BAG than controls. Higher BAG was associated with greater disease severity, neuroimaging markers – most prominently peak width of skeletonized mean diffusivity – and poorer clinical performance. In addition, BAG showed a partial mediation effect in the association between disease stage and cognitive performance.

**DISCUSSION:**

Accelerated brain aging is evident in CADASIL, and the BAG reflects the cumulative microvascular injury burden and may be involved in the pathophysiological pathway linking disease progression to cognitive impairment.

## Highlights

- BAG is significantly higher in CADASIL than in healthy controls.
- BAG increases progressively with advancing disease severity.
- Peak width of skeletonized mean diffusivity independently correlates with BAG.
- Greater BAG is associated with worse global cognition and functional status.
- BAG may be involved in the link between disease stage and cognitive impairment.

## 1 BACKGROUND

Cerebral autosomal dominant arteriopathy with subcortical infarcts and leukoencephalopathy (CADASIL) is the most common inherited form of cerebral small vessel disease (CSVD), predominantly caused by cysteine‐altering variants in the *NOTCH3* gene.^1^, ^2^ Hallmark neuroimaging features of CADASIL include extensive white matter hyperintensities (WMHs), lacunes, and cerebral microbleeds (CMBs).^3^, ^4^ Clinically, *NOTCH3*‐related CSVD is characterized by a progressive course ranging from asymptomatic neuroimaging abnormalities to recurrent strokes, cognitive and gait impairment, and ultimately disability.^5^ Although magnetic resonance imaging (MRI)‐ and functional status‐based staging systems have been proposed,^5^ the factors that govern interindividual variability in disease severity and adverse clinical outcomes remain incompletely understood.^6^, ^7^ In particular, established neuroimaging or clinical markers do not fully explain the marked heterogeneity in clinical trajectories observed among individuals with CADASIL.^8^, ^9^, ^10^

Biological age is conceptualized as an individual's physiological standing relative to their chronological age, serving as a dynamic indicator of functional integrity and age‐related vulnerability that may deviate from time‐based aging.^11^ Recent advances in neuroimaging have enabled brain‐age prediction models that use machine learning applied to large normative MRI datasets to estimate an individual's brain biological age, which provides a global measurement that represents overall brain health across the aging process. The difference between predicted and chronological age, termed brain age gap (BAG), provides a quantitative index of accelerated or delayed brain aging. Neuroimaging‐derived BAG has been applied across a range of neurodegenerative and neuropsychiatric diseases, advancing both clinical applications and mechanistic insights.^17^, ^18^, ^19^, ^20^, ^21^ However, the association between neuroimaging‐defined brain age and CADASIL remains unknown.

In this study, we applied a validated brain‐age prediction model, built upon neuroimaging‐derived age‐related features, to a cohort of individuals carrying cysteine‐altering *NOTCH3* variants and a group of age‐ and sex‐matched healthy controls. We aimed to elucidate the brain aging mechanisms in CADASIL. Specifically, we investigated whether BAG was elevated in CADASIL, how it varied across disease severity, and how it related to neuroimaging features of CSVD, glymphatic function, and white matter microstructural integrity, as well as to adverse clinical outcomes. We hypothesized that patients with CADASIL would exhibit accelerated brain aging that reflected the cumulative burden of CSVD lesions or white matter microstructural damage and represented an intermediate process linking disease stage to cognitive decline.

## 2 METHODS

### 2.1 Standard protocol approvals, registrations, and patient consent

The study was approved by the Institutional Review Board of Taipei Veterans General Hospital (TVGH IRB No. 2017‐02‐008A) according to the guidelines of the Declaration of Helsinki of 1975. Written informed consent was obtained from all participants.

A consecutive series of 153 participants carrying cysteine‐altering *NOTCH3* variants were recruited from the Department of Neurology, Taipei Veterans General Hospital, between December 2014 and November 2024. In addition, 30 age‐ and sex‐matched healthy individuals without non‐synonymous *NOTCH3* variants, scanned using the same MRI protocol during the same period, were enrolled as controls. Participants with *NOTCH3* variants were stratified into three groups (Stage 0‐1, Stage 2, and Stage 3) according to the established *NOTCH3*‐associated small vessel disease (*NOTCH3*‐SVD) staging system.^5^

### 2.2 Clinical evaluation

A structured questionnaire was used to collect demographic data (age, sex, and years of education), smoking habits, and medical history, including stroke (ischemic or hemorrhagic), cognitive impairment, gait disturbance, hypertension, diabetes, and hyperlipidemia. Most participants with *NOTCH3* variants underwent cognitive and clinical assessments, including the Mini‐Mental State Examination (MMSE, *n* = 148)^22^ to evaluate global cognitive function and the Trail Making Test Part A (TMT‐A, *n* = 130)^23^ to assess processing speed. MMSE and TMT‐A scores were converted to age‐ and education‐adjusted *z*‐scores.^24^ Functional status and disability were evaluated using the modified Rankin Scale (mRS, *n* = 153).^25^

### 2.3 Construction of brain‐age prediction model

A brain‐age prediction model was trained, constructed, and validated using T1‐weighted imaging (T1WI) data from an independent cohort of 1482 healthy individuals (age range 18 to 92 years) without major neurologic, psychiatric, or systemic medical conditions, as previously described.^20^ Individual voxel‐wise gray matter volume (GMV) maps were generated using a standard voxel‐based morphometry (VBM) pipeline with high‐dimensional diffeomorphic anatomical registration through exponentiated Lie algebra (DARTEL). The procedure included tissue segmentation, study‐specific template generation, normalization to Montreal Neurological Institute (MNI) space, modulation to preserve tissue volume, and 6‐mm full width at half maximum (FWHM) Gaussian smoothing. GMV features were extracted from 442 brain regions, comprising 400 cortical regions, 14 subcortical regions, and 28 cerebellar regions. These 442 regional GMVs served as inputs for a global brain‐age prediction model using support vector regression (SVR) with a radial basis function (RBF) kernel as implemented in Scikit‐learn (version 1.6; https://scikit‐learn.org/stable/). To address the systematic bias inherent in brain‐age models, specifically the overestimation in younger subjects and underestimation in older ones known as the “regression‐to‐the‐mean” phenomenon,^26^, ^27^ we also implemented an age‐bias correction procedure. Specifically, a linear regression was fitted to the training dataset to model the relationship between chronological age and predicted brain age. The resulting parameters were then applied to adjust the brain‐age estimates for both the CADASIL and control groups.^28^ Model accuracy was assessed using mean absolute error (MAE) and coefficient of determination (*R* ^2^), demonstrating high predictive performance (*r* = 0.90, *R* ^2^ = 0.81, MAE = 6.46 years).^20^

**RESEARCH IN CONTEXT**

1. **Systematic review**: We searched PubMed and Google Scholar for studies evaluating neuroimaging biomarkers in CADASIL. While prior research characterized subcortical and white matter lesions, how genetically driven vascular insults translate into global brain aging remains unclear. Although the neuroimaging‐derived BAG has been examined in other neurodegenerative and cerebrovascular diseases, its relevance to disease progression, severity, and outcomes in CADASIL has not been established.
2. **Interpretation**: CADASIL patients exhibit significantly higher BAGs, with stepwise increases across *NOTCH3*‐SVD stages. Greater BAG correlates with poorer cognitive and functional outcomes and mediates the relationship between disease stage and cognitive impairment. These findings suggest that CADASIL is associated with accelerated brain aging, potentially reflecting the downstream effects of cumulative subcortical vascular injury on cognition.
3. **Future directions**: Longitudinal studies and larger, genetically diverse cohorts are needed to evaluate BAG as a marker of disease progression and to assess the generalizability of these findings.

### 2.4 Brain MRI protocols, analysis, and image‐derived marker extraction in study cohort

All participants, including both individuals with *NOTCH3* variants and healthy controls, underwent brain MRI on a 3T scanner (SIGNATM, GE Healthcare, Milwaukee, WI, USA). T1WIs (slice thickness = 1 mm; repetition time = 7.1 ms; echo time = 2.7 ms; inversion time = 450 ms) were acquired to evaluate GMV, total intracranial volume (TIV), and lacunar lesions, characterized as parenchymal defects with signal intensity similar to cerebrospinal fluid and a diameter ranging from 3 to 15 mm.^29^ High‐resolution T2 fluid‐attenuated inversion recovery (FLAIR; slice thickness = 1 mm; repetition time = 6000 ms; echo time = 118 ms; inversion time = 1870 ms) images were acquired to assess WMH, and the WMH fraction was calculated as the percentage of WMH volume relative to TIV. Detailed descriptions of imaging quality control and tissue segmentation/quantification procedures are provided below. We used the previously proposed analytical framework to extract imaging features for each patient.^20^, ^30^, ^31^ Before the image pre‐processing, an experienced neurologist (C.P. Chung) visually examined all MRI scans to exclude participants with organic brain disorders (e.g., brain tumor) or insufficient image quality scans (e.g., severe motion artifacts). All analyses were performed using Statistical Parametric Mapping (SPM12, version 7771; Wellcome Centre for Human Neuroimaging, University College London, UK) and MATLAB R2021a (MathWorks, Natick, MA, USA) with default settings. The pre‐processing pipeline was as follows. (1) Using the Lesion Segmentation Toolbox (LST, version 3.0.0), individual T2 FLAIR images were affine‐registered to the corresponding T1WI images, generating WMH probability maps in native T1 space and corresponding lesion‐filled T1 images. (2) A center‐of‐mass approach was applied to automatically reorient individual lesion‐filled T1WI images to minimize subsequent registration error. (3) Lesion‐filled T1WI images were corrected for intensity non‐uniformity and segmented into tissue probability maps (GM, white matter, and cerebrospinal fluid) using the SPM12 “Segment” module, followed by rigid alignment to MNI space. To improve the anatomical plausibility of subcortical segmentation, enhanced tissue probability maps were used instead of the default SPM12 priors. (4) Using the DARTEL approach, individual GM segments were spatially normalized to MNI space by applying the study‐specific template derived from the independent training cohort, and corresponding flow fields were estimated. (5) The global tissue volumes, including GMV, WMH, and TIV, were calculated in MNI space. (6) The resulting modulated GMV maps were smoothed with a 6‐mm FWHM isotropic Gaussian kernel, and voxels with GM probability < 0.2 were excluded. All pre‐processed images were visually inspected at each step to ensure pre‐processing accuracy and exclude cases with segmentation or normalization errors.

Susceptibility‐weighted imaging (SWI; slice thickness = 2 mm; repetition time = 42.3 ms; echo time = 25.4 ms) was applied to detect CMBs, defined as hypointense foci less than 10 mm in diameter.^32^

Diffusion tensor imaging (DTI; *b* = 1000 s/mm^2^; repetition time = 9500 ms; echo time = 81.5 ms) was performed with diffusion gradients applied in 64 directions. The DTI along the perivascular space (DTI‐ALPS) index was calculated from mean diffusivity (MD) in projection and association fibers at the level of the lateral ventricle, where perivascular spaces predominantly run along the *x*‐axis.^33^ The index was defined as the ratio of MD in the *x*‐direction within these fibers to that in the orthogonal directions, with the *y*‐direction for projection fibers and the *z*‐direction for association fibers. To account for hemispheric variation, the index was computed separately for each hemisphere and then averaged across sides to yield a single measure for each participant.

Peak width of skeletonized mean diffusivity (PSMD) is an established imaging marker of CSVD that reflects global white matter microstructural alterations.^34^ It is defined as the difference between the 95th and 5th percentiles of voxel‐wise MD values within the white matter skeleton. To obtain PSMD, diffusion‐weighted images were first corrected for eddy‐current distortions and head motion, and individual MD maps were generated. A white matter skeleton was then created using tract‐based spatial statistics, onto which voxel‐wise MD values were projected. The 5th and 95th percentile values were automatically extracted with a dedicated shell script (http://www.psmd‐marker.com/) to compute the final PSMD value for each participant.

For each participant, BAG was derived from our established brain‐age prediction model described previously by subtracting chronological age from predicted brain age.^20^ A positive BAG reflects accelerated brain aging, and these values were subsequently used for statistical analyses.

### 2.5 Statistical analyses

All analyses were performed using SPSS software (version 22.0; IBM). A two‐tailed *p* < 0.05 was considered statistically significant. Continuous variables were expressed as mean ± standard deviation (SD) with range, and categorical variables as number (percentage). Group comparisons of continuous variables were conducted using the independent *t*‐test or Mann–Whitney *U* test. For comparisons involving more than two groups, one‐way analysis of variance (ANOVA) was utilized. Categorical variables were compared with the chi‐squared test or Fisher's exact test. Analysis of covariance (ANCOVA), adjusted for relevant covariates, was used to compare imaging markers across control participants and individuals with *NOTCH3* variants.

In addition to sex, education, and TIV adjustment, all analyses involving the BAG were adjusted for chronological age and age^2^ to account for the well‐known age‐dependent and non‐linear bias inherent in brain‐age prediction models, as well as the non‐linear trajectory of brain aging.^35^

Associations between BAG and clinical or neuroimaging variables were examined using linear regression models. Both univariate and multivariate analyses were conducted. Given the potential collinearity among CSVD‐related imaging markers, multicollinearity diagnostics were performed.

Mediation analyses were conducted to examine whether BAG mediates the association between *NOTCH3*‐SVD stage (independent variable) and cognitive performance (dependent variables, including MMSE and processing speed). Indirect effects were estimated using a bootstrapping approach with 5000 resamples, and the proportion mediated (*P*m) was calculated. All mediation models were adjusted for age, age^2^, sex, education, and TIV. Additional mediation analyses would be conducted as hypothesis‐driven analyses to explore potential pathways.

Sensitivity analyses were performed to assess the robustness of the findings. First, subgroup analyses were conducted according to *NOTCH3* p.R544C status and epidermal growth factor‐like repeat (EGFr) domain risk profiles.^36^ Interaction effects were evaluated by including product terms in regression models. Second, analyses restricted to the predominant genetic subgroup (p.R544C carriers or medium‐risk EGFr domain) were conducted. Third, matched analyses using a 1:1 age‐, sex‐, and education‐matched subcohort were performed to minimize potential confounding

In addition, to explore the longitudinal relevance of BAG, an exploratory longitudinal analysis was conducted in a subset of participants with follow‐up data. Linear regression models were used to assess the association between baseline BAG and annual changes in neuroimaging markers.

## 3 RESULTS

### 3.1 Demographic, clinical, and neuroimaging characteristics of study participants

An overview of the participants’ demographics and clinical features is provided in Table 1. A total of 153 individuals carrying *NOTCH3* cysteine‐altering variants were recruited, with a mean age of 58.1 ± 14.4 years; 78 (51.0%) were women. The most prevalent variant was *NOTCH3* p.R544C (85.0%), with four homozygous and 126 heterozygous carriers, whereas other variants were individually rare and are summarized in Table S1. Among these individuals, 59 (38.6%) had a history of stroke, 60 (39.2%) exhibited cognitive decline, and 39 (25.5%) presented with gait abnormalities. Comparisons of vascular risk factors, clinical manifestations, and neuroimaging markers across genetic variant subtypes (*NOTCH3* p.R544C vs non‐p.R544C) and EGFr domain risk groups (high risk vs medium risk) are presented in Tables S2, S3.

**TABLE 1:** Demographic, cardiovascular risk factors, and neuroimaging markers in individuals with *NOTCH3* variants and control.

Demographic variabls	Control (*n* = 30)	Individuals with *NOTCH3* variants (*n* = 153)	*p*
Men	13 (43.3%)	75 (49.0%)	0.569^*^
Age at exam, years	56.43 ± 12.65	58.08 ± 14.38	0.561^*^
Education, years	17.13 ± 4.26	13.02 ± 4.27	<0.001^*^
**Cardiovascular risk factors**			
Hypertension	9 (30.0%)	53 (34.6%)	0.623^*^
Diabetes	0 (0%)	16 (10.5%)	0.078^*^
Hyperlipidemia	7 (23.3%)	49 (32.0%)	0.345^*^
Smoking	4 (13.3%)	35 (22.9%)	0.331^*^
**Clinical manifestations**			
Stroke	–	59 (38.6%)	–
Cognitive impairment	–	60 (39.2%)	–
Gait disturbance	–	39 (25.5%)	–
**Neuroimaging markers**			
WMH fraction, %	0.07 ± 0.17	2.07 ± 1.88	<0.001^†^
Lacune numbers	0.10 ± 0.40	9.90 ± 15.46	<0.001^†^
CMB counts	0.17 ± 0.53	16.33 ± 29.94	<0.001^†^
DTI‐ALPS index	1.43 ± 0.14	1.29 ± 0.18	<0.001^†^
PSMD, 10^−4^mm^2^/s	2.30 ± 0.39	3.78 ± 1.70	<0.001^†^
Brain age gap, years	1.14 ± 8.65	8.10 ± 10.27	0.004^‡^

*Note*: Values are presented as mean ± standard deviation or counts (%).

Abbreviations: CMB, cerebral microbleed; DTI‐ALPS, diffusion tensor image analysis along the perivascular space; PSMD, peak width of skeletonized mean diffusivity; WMH, white matter hyperintensity.

* values were obtained from Student's *t*‐test, Mann‐Whitney U test, chi‐squared test, or Fisher's exact test.

^†^ *p* values were obtained from analysis of covariance, adjusting for age and sex

^‡^ *p* values were obtained from analysis of covariance, adjusting for age, age^2^, sex, education, and total intracranial volume.

For comparison, 30 age‐ and sex‐matched healthy controls without *NOTCH3* pathogenic variants were included (mean age: 56.4 ± 12.7 years; 56.7% women). Compared with controls, individuals with *NOTCH3* variants had significantly lower educational attainment, whereas the prevalence of diabetes showed a non‐significant trend toward being higher. They also exhibited a greater burden of CSVD‐related imaging abnormalities. In addition, individuals carrying *NOTCH3* variants had impaired glymphatic function and white matter microstructural integrity, as reflected by lower DTI‐ALPS index values and higher PSMD values, respectively.

### 3.2 BAG in individuals with *NOTCH3* variants and control groups

**FIGURE 1:**
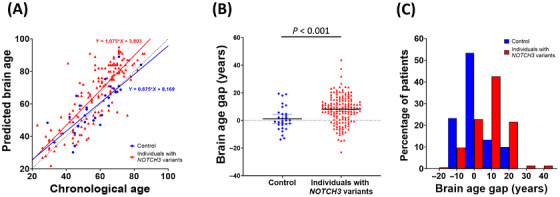
Comparison of brain age and brain age gap (BAG) between individuals with *NOTCH3* variants and controls. (A) Correlation between predicted brain age and chronological age in the control group (blue circles) and individuals with *NOTCH3* variants (red triangles). The red line represents the regression for the *NOTCH3* group, and the blue line represents the control group. The black dashed line indicates equality between predicted brain age and chronological age. (B) Comparison of BAG between control group and individuals with *NOTCH3* variants. The horizontal lines represent the mean and 95% confidence interval. Statistical significance was determined using analysis of covariance adjusted for age, age^2^, sex, education, and total intracranial volume, with the threshold for significance set at a two‐tailed *p *< 0.05. (C) Histogram of BAG in controls (blue bars) and individuals with *NOTCH3* variants (red bars), shown in 10‐year intervals.

The regression slope between chronological and predicted brain age was steeper in individuals with *NOTCH3* variants (*β* = 1.075) than in controls (*β* = 0.875), indicating a more rapid increase in predicted brain age with advancing age (Figure 1A). After adjustment for chronological age, age^2^, sex, education, and TIV, individuals with *NOTCH3* variants exhibited significantly larger BAG values compared with controls (8.10 ± 10.27 vs 1.14 ± 8.65; *p* = 0.004; Figure 1B,C).

**FIGURE 2:**
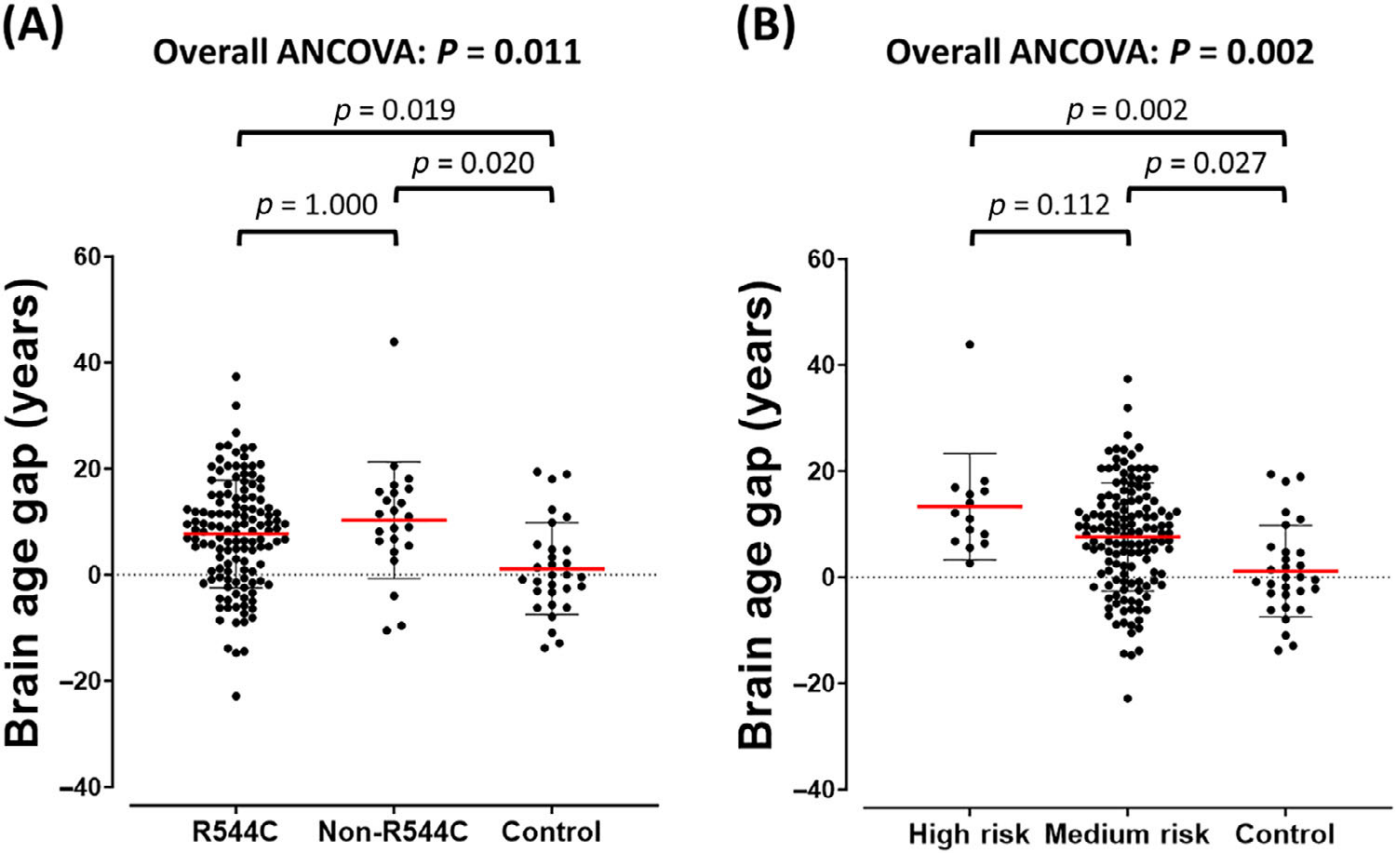
Brain age gap (BAG) across CADASIL subtypes and controls. (A) BAG stratified by *NOTCH3* variant subtype (p.R544C vs non‐p.R544C) and control group. BAG did not differ significantly between CADASIL subtypes, whereas both subgroups exhibited significantly higher BAG compared with controls. (B) BAG stratified by EGFr domain‐defined risk groups (high‐risk vs medium‐risk) and control group. BAG was comparable between EGFr risk groups within CADASIL, but both groups showed significantly higher BAG than controls. The scatter plot displays individual data points, with the red horizontal lines representing the mean and the black error bars the standard deviation. Statistical significance was assessed using analysis of covariance adjusted for age, age^2^, sex, education, and total intracranial volume, followed by post hoc pairwise comparisons (Bonferroni test). A two‐tailed *p* < 0.05 was considered statistically significant.

To evaluate whether the observed elevation in BAG in CADASIL was consistent across disease subtypes, we performed stratified analyses based on *NOTCH3* variant subtypes (p.R544C vs non‐p.R544C) and EGFr domain risk status. Vascular risk profiles differed across subgroups (Table S2, S3): Hypertension was more prevalent among p.R544C carriers than non‐p.R544C carriers, whereas the high‐risk EGFr domain group showed a lower prevalence of hypertension and hyperlipidemia compared with the medium‐risk group. Heterogeneity in neuroimaging CSVD burden was also observed (Table S2, S3). Non‐p.R544C carriers demonstrated greater WMH burden and higher lacune counts, while high‐risk EGFr domain variants were associated with more severe CSVD features, including higher WMH fraction, lacune numbers, and PSMD. Despite these variations, BAG did not differ significantly between subgroups within CADASIL; importantly, in post hoc comparisons with controls, all CADASIL subgroups consistently exhibited significantly higher BAG, indicating that BAG elevation represents a consistent disease‐related feature in CADASIL rather than a subtype‐specific effect (Figure 2).

Although education was adjusted for in the BAG comparisons between CADASIL and controls, we performed an additional sensitivity analysis to further mitigate potential bias arising from imbalanced educational attainment. Specifically, a 1:1 matched subcohort of 30 individuals with *NOTCH3* variants and 30 controls was constructed, balanced for age, sex, and education (Table S4). In this matched analysis, individuals with *NOTCH3* variants continued to exhibit significantly higher BAG compared with controls (6.74 ± 9.86 vs 1.14 ± 8.65 years; *p *= 0.023).

### 3.3 Factors associated with BAG in CADASIL

To identify factors associated with increased BAG, linear regression analyses were performed among individuals with *NOTCH3* variants, adjusting for age, age^2^, sex, education, and TIV (Table 2). In univariate analysis, hypertension, higher PSMD, larger WMH fraction, higher lacune numbers, increased CMB counts, and lower DTI‐ALPS index were each associated with larger BAG. When these variables were included simultaneously in multivariable regression models, PSMD remained significantly associated with BAG (*β* = 3.483, 95% CI, 1.266 to 5.700; *p* = 0.002), whereas the associations with other CSVD‐related imaging measures were attenuated.

**TABLE 2:** Factors related to increased brain age gap in individuals with *NOTCH3* variants.

	Univariate regression analysis		Multivariate regression analysis	
Variables	*β* (95% CI)	*p* ^*^	*β* (95% CI)	*p* ^*^
High risk‐EGFr domains	4.075 (−3.728, 11.877)	0.303	–	–
Hypertension	4.174 (0.113, 8.235)	0.044	1.791 (−1.635, 5.217)	0.303
Diabetes	5.268 (−0.820, 11.355)	0.089	–	–
Hyperlipidemia	−1.169 (−5.154, 2.817)	0.563	–	–
Smoking	−0.485 (−5.338, 4.368)	0.844	–	–
DTI‐ALPS index	−28.376 (−40.677, −16.076)	<0.001	−11.452 (−23.163, 0.259)	0.055
PSMD, 10^−4^mm^2^/s	4.448 (3.385, 5.510)	<0.001	3.483 (1.266, 5.700)	0.002
WMH fraction, %	3.061 (1.988, 4.135)	<0.001	0.252 (−1.294, 1.797)	0.748
Lacune numbers	0.273 (0.164, 0.383)	<0.001	0.0002 (−0.135, 0.135)	0.998
CMB counts	0.169 (0.106, 0.231)	<0.001	0.017 (−0.067, 0.101)	0.684

Abbreviations: CI, confidence interval; CMB, cerebral microbleed; DTI‐ALPS, diffusion tensor image analysis along the perivascular space; EGFr, epidermal growth factor‐like repeat; PSMD, peak width of skeletonized mean diffusivity; WMH, white matter hyperintensity.

* All models were adjusted for age, age2, sex, education, and total intracranial volume.

Collinearity diagnostics showed moderate intercorrelation among imaging variables, with a variance inflation factor of 6.801 for PSMD and <5 for other markers. To further evaluate the stability of the model, hierarchical regression analysis was performed. The addition of PSMD significantly improved model performance, with an incremental *R* ^2^ change of 0.048 (*p* = 0.002). This suggests that PSMD contributes additional explanatory value for BAG beyond other CSVD markers included in the model.

To evaluate the robustness of the association between PSMD and BAG, we performed a series of sensitivity analyses, including restriction to *NOTCH3* p.R544C carriers and stratification by genetic subtype and EGFr domain – defined risk profiles (Tables S5–S7). In the analysis restricted to p.R544C carriers, PSMD remained significantly associated with BAG in multivariable models, whereas other CSVD‐related imaging markers were not (Table S5). In analyses stratified by p.R544C status (Table S6), higher PSMD was associated with greater BAG in p.R544C carriers, whereas the association was attenuated and not statistically significant in non‐p.R544C carriers. However, there was no significant interaction between PSMD and p.R544C status (*p* for interaction = 0.923), suggesting that the association between PSMD and BAG was not significantly modified by variant subtype. Similarly, in analyses stratified by EGFr domain risk status (Table S7), higher PSMD was associated with greater BAG in the medium‐risk group but not in the high‐risk group. No significant interaction was observed between PSMD and EGFr domain risk (*p* for interaction = 0.346), indicating that the relationship between PSMD and BAG was also consistent across EGFr domain risk groups. Together, these findings indicate that the association between PSMD and BAG is robust and not driven by rare variant composition or significantly modified by genetic subtype or EGFr domain risk status in CADASIL. However, the relatively small sample sizes in the non‐p.R544C and high‐risk EGFr subgroups may have limited statistical power, and the consistency of these findings in these subgroups warrants further investigation in larger cohorts. Notably, significant interactions were observed between lacune burden and both p.R544C status (*p* for interaction = 0.003; Table S6) and EGFr domain risk (*p* for interaction < 0.001; Table S7), suggesting potential heterogeneity in the relationship between focal structural lesion burden and BAG across genetic and domain‐defined risk subgroups. These findings should be interpreted with caution and require confirmation in future studies.

### 3.4 Progressive increase in BAG across CADASIL stages

**FIGURE 3:**
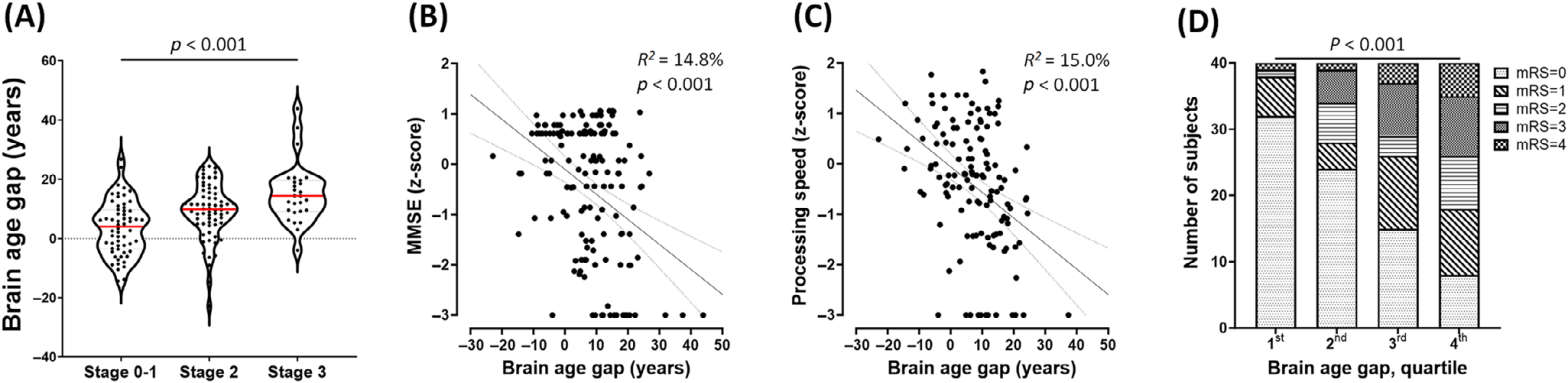
Associations between brain age gap (BAG) and clinical characteristics in individuals with *NOTCH3* variants. (A) Comparison of BAG across different clinical severities (Stage 0‐1, Stage 2, and Stage 3) stratified by *NOTCH3*‐SVD staging system. The plot displays individual data points and the distribution (violin plot). The red horizontal line represents the median, and the dotted lines represent the quartiles. Statistical significance was determined using one‐way ANOVA. (B and C) Association between BAG and Mini‐Mental State Examination (MMSE) *z*‐scores, as well as between BAG and processing speed (Trail Making Test Part A) *z*‐scores. Solid lines represent linear regression fits with 95% confidence intervals (dotted lines). *R* ^2^ denotes the coefficient of determination; the *p* value was derived from regression analyses adjusted for age, age^2^, sex, education, and total intracranial volume. (D) Distribution of modified Rankin scale (mRS) scores (ranging from 0 to 4) across BAG quartiles (first to fourth quartiles) in individuals with *NOTCH3* variants. Differences in mRS distributions across quartiles were evaluated using the Kruskal‐Wallis H test. The *y*‐axis represents the number of subjects in each mRS category. A two‐tailed *p* < 0.05 was considered statistically significant.

In subgroup analysis, individuals with *NOTCH3* variants were stratified into *NOTCH3*‐SVD Stage 0‐1, Stage 2, and Stage 3, and their demographic, clinical, and neuroimaging information was detailed in Table S8. Clinical manifestations, including stroke episodes, cognitive impairment, and gait disturbance, became progressively more severe with advancing disease stage. BAG values also increased across stages, from 3.54 ± 9.13 years in Stage 0‐1 to 9.31 ± 9.34 years in Stage 2 and 15.27 ± 10.19 years in Stage 3 (*p *< 0.001; Figure 3A). Similar stage‐dependent trends were observed for other CSVD‐related imaging markers, including the DTI‐ALPS index, PSMD, WMH fraction, lacune number, and CMB count (Table S8).

### 3.5 Associations between BAG and clinical outcomes in CADASIL

In the overall CADASIL cohort, which included individuals across a range of disease stages, higher BAG values were significantly associated with worse cognitive performance. BAG was inversely correlated with MMSE *z*‐scores (*β* = −0.050, 95% CI: −0.069 to −0.030; *R* ^2^ = 14.8%; *p* < 0.001; Figure 3B) and processing speed *z*‐scores (*β* = −0.051, 95% CI: −0.072 to −0.030; *R* ^2^ = 15.0%; *p* < 0.001; Figure 3C).

The association between BAG and functional status was further examined using the mRS. Higher BAG quartiles were associated with a shift toward greater functional disability, with an increasing proportion of individuals exhibiting greater mRS scores (mRS score > 1) across ascending BAG categories (Figure 3D).

### 3.6 The potential mediating role of BAG in associations between *NOTCH3*‐SVD stages and cognitive performance

**FIGURE 4:**
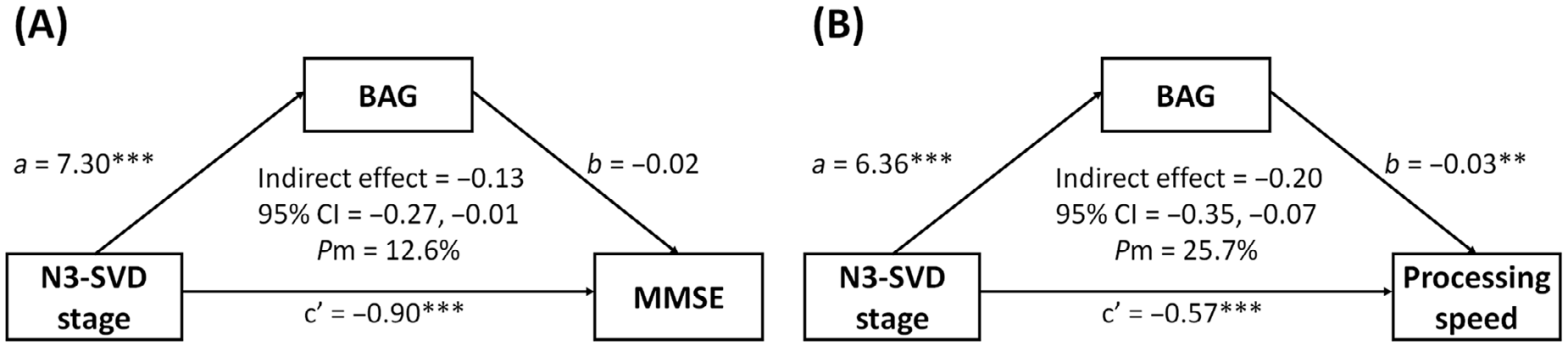
Mediation models illustrating relationship between *NOTCH3*‐SVD (N3‐SVD) stage, brain age gap (BAG), and cognitive performance in individuals with *NOTCH3* variants. The mediation analysis was conducted using the process macro for SPSS (Model 4) to evaluate the mediating effect of BAG on the relationship between the *NOTCH3*‐SVD staging system and cognitive outcomes, including (A) Mini‐Mental State Examination (MMSE) scores and (B) processing speed (Trail Making Test Part A). In each model, the *NOTCH3*‐SVD stage serves as the independent variable, BAG acts as the mediator, and cognitive scores represent the clinical outcomes. The path coefficients *a* and *b* represent the indirect effects, while *c*’ represents the direct effect after adjusting for age, age^2^, sex, education, and total intracranial volume. The indirect effect, its 95% confidence interval (CI), and the percentage of mediation (*P*m, calculated as the ratio of the indirect effect to the total effect) are provided for each model. Statistical significance was assessed using the bootstrapping method with 5000 resamples, with mediation effects considered significant if the 95% CI did not include zero; ^*^ *p* < 0.05, ^**^ *p* < 0.01, ^***^ *p* < 0.001.

To examine whether BAG was involved in the association between disease severity and cognitive performance, mediation analyses were conducted with *NOTCH3*‐SVD stage as the independent variable, adjusting for age, age^2^, sex, education, and TIV. The indirect mediating effect of BAG on the association between *NOTCH3*‐SVD stage and MMSE was significant (−0.13, 95% CI: −0.27 to −0.01, *P*m = 12.6%; Figure 4A). A larger indirect mediating effect was observed for processing speed (−0.20, 95% CI: −0.35 to −0.07, *P*m = 25.7%; Figure 4B). In both models, the direct effects of the *NOTCH3*‐SVD stage on cognitive outcomes remained significant after inclusion of BAG, indicating partial mediation, with a greater proportion mediated for processing speed than for global cognition.

Notably, when PSMD was utilized as the primary independent variable, mediation analysis showed that BAG did not significantly mediate the relationship between white matter microstructural damage and cognitive performance. Specifically, the indirect effect of BAG was not significant for either MMSE (indirect effect = 0.05, 95% CI = −0.04 to 0.15) or processing speed (indirect effect = −0.01, 95% CI = −0.11 to 0.08) (Figure S1). This suggests that white matter microstructural disruption and gray matter‐related brain aging contribute to cognitive impairment through partially distinct or additive mechanisms, rather than a simple linear cascade.

Sensitivity analyses were performed in the major genetic subgroup since models for non‐p.R544C carriers and the high‐risk EGFr domain group were not feasible due to limited sample size. In p.R544C carriers, BAG remained a significant statistical mediator for both MMSE (indirect effect = −0.17, *P*m = 17.9%) (Figure S2A) and processing speed (indirect effect = −0.22, *P*m = 27.1%) (Figure S2B), consistent with a stable mediating association. In the medium‐risk EGFr domain subgroup, the statistical mediating effect of BAG on MMSE was not significant (Figure S2C); however, BAG remained a statistical mediator for processing speed (indirect effect = −0.18, *P*m = 23.1%; Figure S2D). These findings indicate that BAG shows a consistent mediating association between disease severity and cognitive impairment, with a more pronounced pattern in processing speed.

### 3.7 Association of baseline BAG with longitudinal changes in neuroimaging markers

To explore the potential relevance of the brain‐age framework in longitudinal change, we conducted an exploratory analysis in a subset of participants (*n* = 29) with follow‐up durations ranging from 12 to 56 months. We examined the associations between baseline BAG and annual changes in neuroimaging markers (Table S9). Baseline BAG was significantly associated with greater annual increases in PSMD (*β* = 0.013, 95% CI: 0.004 to 0.023; *R* ^2^ = 29.6%; *p* = 0.007; Figure S3). In contrast, no significant associations were observed between baseline BAG and changes in WMH fraction, lacune number, CMB count, or DTI‐ALPS index. Given the limited sample size and observational design, these findings should be interpreted as exploratory. The observed association with PSMD suggests a potential link between baseline brain aging and subsequent white matter microstructural changes, although the directionality and underlying mechanisms remain to be clarified.

## 4 DISCUSSION

This study applied a brain‐age prediction model to characterize brain aging in CADASIL. By quantifying the gap between imaging‐derived brain age and chronological age, this approach captures global gray matter aging beyond conventional neuroimaging markers. We found that increased BAG was a key feature of CADASIL, closely associated with *NOTCH3*‐SVD stage, imaging markers of CSVD burden, and clinical performance. These findings suggest that accelerated brain aging reflects disease severity and the cumulative burden of microvascular injury, particularly white matter microstructural disruption, and may contribute to brain dysfunction in CADASIL.

While increased imaging‐defined brain age has been reported in several neurological diseases,^18^, ^19^, ^20^, ^21^, ^37^, ^38^ its presence and clinical relevance in CADASIL have not been established. To our knowledge, this is the first study to systematically characterize the brain‐age profile in CADASIL. As a genetically defined human model of CSVD‐related brain injury, CADASIL provides a unique framework for examining how chronic vascular pathology influences brain aging. In this study, we found that individuals with CADASIL exhibited accelerated brain aging, reflected by an increased BAG, compared with age‐ and sex‐matched healthy controls. It is important to note that brain‐age prediction models are subject to a well‐recognized age‐related bias, often referred to as regression to the mean, whereby predicted brain age tends to be overestimated in younger individuals and underestimated in older individuals when model accuracy is imperfect. To account for this, we applied an established age‐bias correction procedure based on the relationship between predicted and chronological age in the training dataset. After correction, the relationship between predicted brain age and chronological age in controls approximated the expected identity pattern, whereas the CADASIL group exhibited a steeper slope (Figure 1A). This pattern suggests that the discrepancy between predicted and chronological age becomes more pronounced with advancing age in CADASIL.

Most neuroimaging studies in CADASIL have primarily focused on subcortical CSVD lesions and white matter microstructural integrity, with comparatively limited investigation of gray matter involvement. Early volumetric work demonstrated global brain atrophy without distinguishing gray and white matter compartments,^39^ while subsequent voxel‐based morphometry and cortical thickness studies – typically with small sample sizes (*n* = 14 to 22) – reported region‐specific cortical abnormalities that remained fragmented and lacked integration into a system‐level framework.^40^, ^41^ In this context, our GMV‐derived brain‐age model provides a complementary perspective by capturing subtle, spatially distributed gray matter changes across the whole brain. Building on this, our findings further link vascular pathology to neuroimaging‐defined brain aging in CADASIL. Among multiple CSVD markers, PSMD – a sensitive indicator of diffuse white matter microstructural damage – showed the strongest and independent association with BAG, although collinearity among imaging markers warrants cautious interpretation. The predominance of PSMD suggests that widespread white matter disruption, rather than focal lesion burden alone, plays a central role in accelerated global gray matter aging. Prior evidence suggested that white matter tracts underpinned large‐scale brain connectivity and that diffuse microstructural damage could disrupt long‐range communication and network integration, leading to secondary remote cortical changes.^42^, ^43^ Accordingly, subcortical ischemia may induce cortical alterations in anatomically distant regions through the degeneration of network connecting pathways. In line with these proposed mechanisms, our results suggest that GMV‐derived BAG represents an integrative, system‐level manifestation of network‐wide disruption driven by chronic vascular injury, rather than localized structural damage alone.

Higher BAG was associated with worse global cognition, slower processing speed, and greater functional disability. Mediation analyses further indicated that BAG linked *NOTCH3*‐SVD stage to cognitive performance, with a stronger effect for processing speed – a hallmark domain of subcortical dysfunction in CADASIL that often precedes global decline.^44^, ^45^ These findings suggest that BAG may serve as an intermediate marker linking cumulative vascular injury to cognitive dysfunction, particularly in domains dependent on white matter integrity.

Prior longitudinal evidence established that elevated BAG was associated with future cognitive decline and disease progression across a range of neurodegenerative conditions.^46^, ^47^, ^48^ To explore whether a similar pattern may be present in CADASIL, we conducted a preliminary longitudinal analysis in a subset of patients. Among the neuroimaging markers evaluated, only the annual change in PSMD was significantly associated with baseline BAG. These findings suggest that higher baseline BAG may be linked to subsequent deterioration in white matter microstructure, further reinforcing the close association between BAG and white matter integrity. However, BAG did not mediate the PSMD–cognition relationship, suggesting it reflects an integrative marker of global brain vulnerability rather than a direct pathway. The interaction between BAG and PSMD remains unclear and warrants investigation in larger longitudinal, multimodal studies.

An important consideration in interpreting our findings is the predominance of the p.R544C variant in this cohort. Subgroup and sensitivity analyses showed that accelerated brain aging was consistent across genotypes, with no differences between p.R544C and non‐p.R544C carriers or EGFr risk groups. Although non‐p.R544C carriers exhibited a higher burden of structural lesions, including greater WMH fraction and lacune counts, the overall patterns of association between BAG and imaging markers were largely consistent across genotypes. In particular, the relationship between PSMD and BAG remained robust and was not significantly modified by p.R544C status. Consistent findings were also observed in subgroup mediation analyses. Notably, a significant interaction was observed for lacune burden, suggesting potential heterogeneity in the contribution of focal lesion burden to brain aging across genotypes. However, given the limited sample size of non‐p.R544C carriers, this observation requires confirmation in larger cohorts.

Recent literature has highlighted a potential divergence between brain‐age model accuracy and its clinical utility.^16^ Although our model showed robust predictive performance, the primary aim of this study was not to validate BAG as a clinical biomarker but to use it as an imaging‐derived construct to provide insight into the mechanisms of neurodegeneration in CADASIL. Nevertheless, BAG may have potential clinical relevance. Before it can be considered for use as a clinical biomarker or trial endpoint, several steps are required, including validation of its longitudinal sensitivity to disease progression and treatment effects, assessment of reproducibility across MRI platforms, and establishment of clinically meaningful thresholds (e.g., cutoff values or minimal clinically important differences) in relation to functional outcomes.

From a methodological perspective, we used a region‐of‐interest (ROI)‐based SVR model for practical and conceptual reasons. T1‐weighted imaging was the only sequence consistently available across sites, maximizing sample size and generalizability. SVR with region‐based features provides reliable performance for moderate datasets and is robust to overfitting.^49^ Importantly, ROI‐based models offer anatomical interpretability, aligning with our mechanistic focus on linking brain age to structural and network‐level changes in CSVD. In contrast, deep learning approaches require larger datasets and do not consistently outperform conventional models.^50^, ^51^ This strategy balances interpretability, robustness, and cross‐cohort applicability.

Several limitations should be acknowledged. First, this was a single‐center study with a predominance of participants of Han Chinese ancestry and a high frequency of the *NOTCH3* p.R544C variant, which may limit generalizability to other ethnic groups and mutation spectra. In addition, the control group was relatively small and had a higher educational level, which may introduce selection bias and influence estimates of BAG differences, although all analyses were adjusted for education. To mitigate this limitation, we applied matching approaches (Table S4), which yielded consistent BAG profiles. Nevertheless, these findings should be interpreted with caution, and validation in larger, more representative cohorts is warranted. Second, the brain‐age prediction model was derived primarily from GMV features. While this approach is well suited to capture remote cortical effects of chronic vascular injury, an underexplored aspect of CADASIL, it does not directly measure white matter microstructural integrity. Future multimodal models integrating diffusion‐based metrics may further refine brain‐age estimates in CADASIL. Third, although a lesion‐filling procedure was applied prior to tissue segmentation to mitigate white matter hyperintensity‐related misclassification, residual bias cannot be entirely excluded, particularly in advanced CADASIL with extensive tissue alteration. Such disease‐related changes may introduce a degree of domain shift relative to the model's training population, potentially influencing gray matter estimation and, consequently, BAG interpretation. Fourth, cognitive tests assessing global cognition function and processing speed may not fully capture other domain‐specific cognitive deficits. Finally, because the mediation analysis was based on cross‐sectional data, the mediating role of BAG should be interpreted as a statistical association rather than evidence of causality. Nevertheless, the observed relationships are consistent with a potential mechanistic pathway linking accelerated brain aging to disease severity, structural alterations, and cognitive impairment in CADASIL.

In conclusion, accelerated brain aging is a key feature of CADASIL. BAG is associated with disease severity and adverse clinical outcomes, reflecting the cumulative impact of CSVD on brain structure. These findings suggest that BAG captures the integrated effects of microvascular injury and may represent a clinically informative marker of disease burden.

## CONFLICT OF INTEREST STATEMENT

The authors declare no conflicts of interest. Author disclosures are available in the Supporting Information.

## CONSENT STATEMENT

All human subjects provided informed consent.

## Supporting information




**Supporting Information**: alz71535‐sup‐0001‐SuppMat

**Supporting Information**: alz71535‐sup‐0002‐SuppMat
