# Supplementary material for: Association of imaging‐defined brain age with disease severity and adverse outcomes in CADASIL
Source: Alzheimers Dement. 2026 Jun 2;22(6):e71535. doi: 10.1002/alz.71535 (PMC13239354; doi:10.1002/alz.71535)
Supplement: Supplementary file 2 — Supporting Information: alz71535‐sup‐0002‐SuppMat [file ALZ-22-e71535-s002.docx]

**Table S1. Distribution of *NOTCH3* variants among study participants**

| *NOTCH3* variant | EGFr domain | Count (n) | Percentage (%) |
| --- | --- | --- | --- |
| C49F | High-risk | 1 | 0.7% |
| R54C | High-risk | 1 | 0.7% |
| S118C | High-risk | 2 | 1.3% |
| R133C | High-risk | 2 | 1.3% |
| R141C | High-risk | 1 | 0.7% |
| C222S | High-risk | 4 | 2.6% |
| C233S | High-risk | 1 | 0.7% |
| R332C | High-risk | 1 | 0.7% |
| R544C^*^ | Medium-risk | 130 | 85.0% |
| R558C | Medium-risk | 1 | 0.7% |
| C977S | Medium-risk | 4 | 2.6% |
| Y1031C | High-risk | 1 | 0.7% |
| Y1069C | Medium-risk | 1 | 0.7% |
| C1250R | Medium-risk | 2 | 1.3% |
| C1261Y | Medium-risk | 1 | 0.7% |
| Total | - | 153 | 100.0% |

Abbreviations: EGFr = epidermal growth factor-like repeat.

^*^Among participants with the R544C variant, 126 (82.4%) were heterozygous and 4 (2.6%) were homozygous.

**Table S2. Clinical and neuroimaging features stratified by *NOTCH3* p.R544C and non-p.R544C variants.**

| Neuroimaging markers | p.R544C  (n = 130) | Non-p.R544C  (n = 23) | Mean difference (95% CI) | Effect size | *P* |
| --- | --- | --- | --- | --- | --- |
| Men | 63 (48.5%) | 12 (52.2%) | - | - | 0.743^‡^ |
| Age at exam, years | 57.87 ± 14.51 | 59.21 ± 13.86 | - | - | 0.725^‡^ |
| Education, years | 13.25 ± 4.01 | 11.70 ± 5.44 | - | - | 0.306^‡^ |
| Cardiovascular risk factors | | | | | |
| Hypertension | 50 (38.5%) | 3 (13.0%) | - | 5.397 (1.430, 20.372)^*^ | 0.018^‡^ |
| Diabetes | 14 (10.8%) | 2 (8.7%) | - | 1.365 (0.281, 6.641)^*^ | 1.000^‡^ |
| Hyperlipidemia | 45 (34.6%) | 4 (17.4%) | - | 2.796 (0.870, 8.991)^*^ | 0.145^‡^ |
| Smoking | 31 (23.8%) | 4 (17.4%) | - | 1.800 (0.511, 6.344)^*^ | 0.599^‡^ |
| Clinical manifestations | | | | | |
| Stroke | 47 (36.2%) | 12 (52.2%) | - | 0.479 (0.178, 1.294)^*^ | 0.146^‡^ |
| Cognitive impairment | 51 (39.2%) | 9 (39.1%) | - | 1.114 (0.355, 3.493)^*^ | 0.993^‡^ |
| Gait disturbance | 31 (23.8%) | 8 (34.8%) | - | 0.544 (0.189, 1.567)^*^ | 0.267^‡^ |
| Neuroimaging markers | | | | | |
| WMH fraction, % | 1.86 ± 1.81 | 3.28 ± 1.86 | -1.31 (-1.95, -0.67) | 0.099^†^ | <0.001^§^ |
| Lacune numbers | 8.58 ± 12.90 | 17.35 ± 24.62 | -8.18 (-14.60, -1.76) | 0.041^†^ | 0.013^§^ |
| CMB counts | 15.94 ± 31.33 | 18.52 ± 20.78 | -1.72 (-14.52, 11.08) | 0.0004^†^ | 0.791^§^ |
| DTI-ALPS index | 1.29 ± 0.19 | 1.26 ± 0.14 | 0.02 (-0.05, 0.09) | 0.003^†^ | 0.517^§^ |
| PSMD, 10^-4^mm^2^/s | 3.69 ± 1.70 | 4.33 ± 1.65 | -0.55 (-1.23, 0.13) | 0.021^†^ | 0.111^§^ |

Abbreviations: CMB = cerebral microbleed; DTI-ALPS = diffusion tensor image analysis along the perivascular space; MMSE = Mini-Mental State Examination; mRS = modified Rankin Scale; PSMD = peak width of skeletonized mean diffusivity; WMH = white matter hyperintensity.

Values are presented as mean ± standard deviation or counts (%).

^*^Effect size is presented as odds ratio (95% confidence interval)

^†^Effect size is presented as eta squared (η^2^)

^‡^*P* values were obtained from the Student’s t test, Mann-Whitney U test, Chi-square test, or Fisher’s exact test.

^§^*P* values were obtained from analysis of covariance, adjusting for age and sex.

^||^*P* values were obtained from analysis of covariance, adjusting for age, age², sex, education, and total intracranial volume.

**Table S3. Clinical and neuroimaging features stratified by *NOTCH3* EGFr domain risk profiles.**

| Neuroimaging markers | High-risk EGFr domain  (n = 14) | Medium-risk EGFr domain  (n = 139) | Mean difference (95% CI) | Effect size | *P* |
| --- | --- | --- | --- | --- | --- |
| Men | 7 (50.0%) | 68 (48.9%) | - | - | 0.939^‡^ |
| Age at exam, years | 55.05 ± 13.33 | 58.38 ± 14.49 | - | - | 0.411^‡^ |
| Education, years | 13.50 ± 4.82 | 12.97 ± 4.23 | - | - | 0.660^‡^ |
| Cardiovascular risk factors | | | | | |
| Hypertension | 1 (7.1%) | 52 (37.4%) | - | 0.141 (0.017, 1.158)^*^ | 0.035^‡^ |
| Diabetes | 0 | 16 (11.5%) | - | - | 0.364^‡^ |
| Hyperlipidemia | 1 (7.1%) | 48 (34.5%) | - | 0.159 (0.020, 1.269)^*^ | 0.038^‡^ |
| Smoking | 2 (14.3%) | 33 (23.7%) | - | 0.453 (0.085, 2.414)^*^ | 0.525^‡^ |
| Clinical manifestations | | | | | |
| Stroke | 7 (50.0%) | 52 (37.4%) | - | 2.598 (0.740, 9.123)^*^ | 0.356^‡^ |
| Cognitive impairment | 5 (35.7%) | 55 (39.6%) | - | 1.445 (0.349, 5.976)^*^ | 0.778^‡^ |
| Gait disturbance | 5 (35.7%) | 34 (24.5%) | - | 2.972 (0.785, 11.250)^*^ | 0.357^‡^ |
| Neuroimaging markers | | | | | |
| WMH fraction, % | 3.67 ± 1.94 | 1.91 ± 1.81 | 2.03 (1.26, 2.80) | 0.155^†^ | <0.001^§^ |
| Lacune numbers | 12.87 ± 30.18 | 8.86 ± 20.14 | 12.35 (4.46, 20.24) | 0.060^†^ | 0.002^§^ |
| CMB counts | 17.21 ± 21.20 | 16.24 ± 30.74 | 3.25 (-12.63, 19.13) | 0.001^†^ | 0.686^§^ |
| DTI-ALPS index | 1.28 ± 0.10 | 1.29 ± 0.19 | -0.06 (-0.15, 0.03) | 0.015^†^ | 0.173^§^ |
| PSMD, 10^-4^mm^2^/s | 4.42 ± 1.77 | 3.73 ± 1.69 | 1.09 (0.22, 1.96) | 0.048^†^ | 0.014^§^ |

Abbreviations: CMB = cerebral microbleed; DTI-ALPS = diffusion tensor image analysis along the perivascular space; EGFr = epidermal growth factor-like repeat; MMSE = Mini-Mental State Examination; mRS = modified Rankin Scale; PSMD = peak width of skeletonized mean diffusivity; WMH = white matter hyperintensity.

Values are presented as mean ± standard deviation or counts (%).

^*^Effect size is presented as odds ratio (95% confidence interval)

^†^Effect size is presented as eta squared (η^2^)

^‡^*P* values were obtained from the Student’s t test, Mann-Whitney U test, Chi-square test, or Fisher’s exact test.

^§^*P* values were obtained from analysis of covariance, adjusting for age and sex.

^||^*P* values were obtained from analysis of covariance, adjusting for age, age², sex, education, and total intracranial volume.

**Table S4. Neuroimaging features and brain age gap in a 1:1 age-, sex-, and education-matched sub-cohort of *NOTCH3* variants and control participants.**

| Variables | Control (n = 30) | Matched individuals with *NOTCH3* variants (n = 30) | *P* |
| --- | --- | --- | --- |
| Men | 13 (43.3%) | 13 (43.3%) | 1.000 |
| Age at exam, years | 56.43 ± 12.65 | 56.66 ± 16.37 | 0.945 |
| Education, years | 17.13 ± 4.26 | 15.60 ± 3.77 | 0.145 |
| WMH fraction, % | 0.07 ± 0.17 | 1.75 ± 1.96 | <0.001^*^ |
| Lacune numbers | 0.10 ± 0.40 | 6.33 ± 10.17 | 0.001^*^ |
| CMB counts | 0.17 ± 0.53 | 11.37 ± 23.27 | 0.010^*^ |
| DTI-ALPS index | 1.43 ± 0.14 | 1.31 ± 0.17 | 0.001^*^ |
| PSMD, 10^-4^mm^2^/s | 2.30 ± 0.39 | 3.57 ± 1.67 | <0.001^*^ |
| Brain age gap, years | 1.14 ± 8.65 | 6.74 ± 9.86 | 0.023^†^ |

Abbreviations: CMB = cerebral microbleed; DTI-ALPS = diffusion tensor image analysis along the perivascular space; PSMD = peak width of skeletonized mean diffusivity; WMH = white matter hyperintensity.

^*^*P* values were obtained from analysis of covariance, adjusting for age and sex.

^†^*P* values were obtained from analysis of covariance, adjusting for age, age², sex, education, and total intracranial volume.

**Table S5. Factors related to increased brain age gap in individuals with *NOTCH3* R544C variant**

| Variables | Univariate regression analysis | | Multivariate regression analysis | |
| --- | --- | --- | --- | --- |
|  | β (95% CI) | *P*^*^ | β (95% CI) | *P*^*^ |
| Hypertension | 4.105 (0.158, 8.053) | 0.042 | 1.113 (-2.540, 4.765) | 0.547 |
| Diabetes | 2.496 (-3.316, 8.308) | 0.397 | - | - |
| Hyperlipidemia | 1.444 (-2.445, 5.334) | 0.464 | - | - |
| Smoking | -0.017 (-4.812, 4.778) | 0.994 | - | - |
| DTI-ALPS index | -30.203 (-43.862, -16.544) | <0.001 | -10.953 (-23.953, 2.047) | 0.098 |
| PSMD, 10^-4^mm^2^/s | 4.617 (3.491, 5.743) | <0.001 | 4.436 (1.742, 7.130) | 0.001 |
| WMH fraction, % | 2.802 (1.615, 3.988) | <0.001 | -0.734 (-2.539, 1.072) | 0.422 |
| Lacune numbers | 0.436 (0.308, 0.564) | <0.001 | 0.062 (-0.176, 0.300) | 0.607 |
| CMB counts | 0.135 (0.080, 0.189) | <0.001 | -0.014 (-0.109, 0.081) | 0.774 |

Abbreviations: CI = confidence interval; CMB = cerebral microbleed; DTI-ALPS = diffusion tensor image analysis along the perivascular space; PSMD = peak width of skeletonized mean diffusivity; WMH = white matter hyperintensity.

^*^All models were adjusted for age, age^2^, sex, education, and total intracranial volume

**Table S6. Sensitivity analysis of the associations between clinical/neuroimaging factors and brain age gap, stratified by *NOTCH3* p.R544C status.**

| Variables | p.R544C (n = 130) | | Non-p.R544C (n = 23) | |  |
| --- | --- | --- | --- | --- | --- |
|  | β (95% CI) | *P*^*^ | β (95% CI) | *P*^*^ | *P* for interaction^†^ |
| Hypertension | 4.105 (0.158, 8.053) | 0.042 | 1.729 (-14.198, 17.656) | 0.821 | 0.901 |
| Diabetes | 2.496 (-3.316, 8.308) | 0.397 | 0.121 (-18.798, 19.040) | 0.989 | 0.590 |
| Hyperlipidemia | 1.444 (-2.445, 5.334) | 0.464 | -10.592 (-23.066, 1.882) | 0.091 | 0.023 |
| Smoking | -0.017 (-4.812, 4.778) | 0.994 | 9.010 (-7.118, 25.137) | 0.254 | 0.060 |
| DTI-ALPS index | -30.203 (-43.862, -16.544) | <0.001 | -15.329 (-68.299, 37.640) | 0.537 | 0.381 |
| PSMD, 10^-4^mm^2^/s | 4.617 (3.491, 5.743) | <0.001 | 1.290 (-3.161, 5.741) | 0.537 | 0.923 |
| WMH fraction, % | 2.802 (1.615, 3.988) | <0.001 | 3.663 (0.194, 7.132) | 0.040 | 0.389 |
| Lacune numbers | 0.436 (0.308, 0.564) | <0.001 | 0.044 (-0.160, 0.247) | 0.655 | 0.003 |
| CMB counts | 0.135 (0.080, 0.189) | <0.001 | 0.072 (-0.216, 0.360) | 0.603 | 0.669 |

Abbreviations: CI = confidence interval; CMB = cerebral microbleed; DTI-ALPS = diffusion tensor image analysis along the perivascular space; PSMD = peak width of skeletonized mean diffusivity; WMH = white matter hyperintensity.

^*^All models were adjusted for age, age^2^, sex, education, and total intracranial volume

^†^*P* for interaction was calculated using the product term between p.R544C status and each independent variable.

**Table S7. Sensitivity analysis of the associations between clinical/neuroimaging factors and brain age gap, stratified by *NOTCH3* EGFr domain risk profiles**

| Variables | High-risk EGFr domain  (n = 14) | | Medium-risk EGFr domain  (n = 139) | |  |
| --- | --- | --- | --- | --- | --- |
|  | β (95% CI) | *P*^*^ | β (95% CI) | *P*^*^ | *P* for interaction^†^ |
| Hypertension | -5.376 (-30.114, 19.362) | 0.623 | 4.583 (0.805, 8.361) | 0.018 | 0.234 |
| Diabetes | - | - | 3.767 (-1.724, 9.258) | 0.177 | - |
| Hyperlipidemia | -5.376 (-30.114, 19.362) | 0.370 | 0.384 (-3.369, 4.137) | 0.840 | 0.370 |
| Smoking | 11.243 (-4.887, 27.373) | 0.143 | 1.065 (-3.592, 5.723) | 0.652 | 0.054 |
| DTI-ALPS index | -17.241 (-90.339, 55.856) | 0.507 | -32.643 (-45.945, -19.341) | <0.001 | 0.294 |
| PSMD, 10^-4^mm^2^/s | 0.688 (-4.650, 6.027) | 0.709 | 4.664 (3.522, 5.807) | <0.001 | 0.346 |
| WMH fraction, % | 3.028 (-1.309, 7.366) | 0.143 | 3.024 (1.865, 4.183) | <0.001 | 0.753 |
| Lacune numbers | -0.018 (-0.255, 0.218) | 0.862 | 0.438 (0.313, 0.564) | <0.001 | <0.001 |
| CMB counts | 0.310 (-0.221, 0.841) | 0.210 | 0.139 (0.085, 0.193) | <0.001 | 0.494 |

Abbreviations: CI = confidence interval; CMB = cerebral microbleed; DTI-ALPS = diffusion tensor image analysis along the perivascular space; EGFr = epidermal growth factor-like repeat; PSMD = peak width of skeletonized mean diffusivity; WMH = white matter hyperintensity.

^*^All models were adjusted for age, age^2^, sex, education, and total intracranial volume

^†^*P* for interaction was calculated using the product term between EGFr domain risk profiles and each independent variable.

**Table S8. Clinical and neuroimaging features of individuals with *NOTCH3* variants**

| Demographic variables | *NOTCH3*‑SVD stage | | | *p*^*^ |
| --- | --- | --- | --- | --- |
|  | Stage 0-1 (n = 60) | Stage 2 (n = 66) | Stage 3 (n = 27) |  |
| Men | 20 (33.3%) | 40 (60.6%) | 15 (55.6%) | 0.007 |
| Age at exam, years | 46.11 ± 12.91 | 65.17 ± 9.25 | 67.33 ± 8.34 | <0.001 |
| Education, years | 15.28 ± 2.52 | 12.03 ± 4.14 | 10.41 ± 5.29 | <0.001 |
| High risk-EGFr domains | 3 (5.0%) | 7 (10.6%) | 1 (3.7%) | 0.354 |
| MMSE, z-score | 0.24 ± 0.67 | -0.34 ± 1.15 | -2.61 ± 0.57 | <0.001 |
| Processing speed, z-score | 0.18 ± 0.79 | -0.50 ± 1.20 | -2.31 ± 0.85 | <0.001 |
| mRS | 0 | 0.99 ± 0.73 | 3.26 ± 0.45 | <0.001 |
| Cardiovascular risk factors | | | | |
| Hypertension | 7 (11.7%) | 29 (43.9%) | 17 (63.0%) | <0.001 |
| Diabetes | 3 (5.0%) | 11 (16.7%) | 2 (7.4%) | 0.087 |
| Hyperlipidemia | 12 (20.0%) | 25 (37.9%) | 12 (44.4%) | 0.031 |
| Smoking | 7 (11.7%) | 20 (30.3%) | 8 (29.6%) | 0.030 |
| Clinical manifestations | | | | |
| Stroke | 0 | 38 (57.6%) | 21 (77.8%) | <0.001 |
| Cognitive impairment | 1 (1.7%) | 36 (54.5%) | 23 (85.2%) | <0.001 |
| Gait disturbance | 0 | 21 (31.8%) | 18 (66.7%) | <0.001 |
| Neuroimaging markers | | | | |
| WMH fraction, % | 0.40 ± 0.94 | 2.87 ± 1.59 | 3.82 ± 1.11 | <0.001 |
| Lacune numbers | 0 | 13.29 ± 18.01 | 23.59 ± 11.65 | <0.001 |
| CMB counts | 0.42 ± 1.39 | 14.82 ± 18.20 | 55.37 ± 47.27 | <0.001 |
| DTI-ALPS index | 1.42 ± 0.18 | 1.22 ± 0.11 | 1.11 ± 0.12 | <0.001 |
| PSMD, 10^-4^mm^2^/s | 2.35 ± 0.49 | 4.37 ± 1.17 | 6.53 ± 1.28 | <0.001 |
| Brain age gap, years | 3.54 ± 9.13 | 9.31 ± 9.34 | 15.27 ± 10.19 | <0.001 |

Abbreviations: CMB = cerebral microbleed; DTI-ALPS = diffusion tensor image analysis along the perivascular space; EGFr = epidermal growth factor-like repeat;

MMSE = Mini-Mental State Examination; mRS = modified Rankin Scale; PSMD = peak width of skeletonized mean diffusivity; WMH = white matter hyperintensity.

Values are presented as mean ± standard deviation or n (%).

^*^*P* values were obtained from one-way analysis of variance, Chi-square test, or Fisher’s exact test.

**Table S9. Association between baseline brain age gap and the annual changes in MRI parameters in the individuals with *NOTCH3* variants**

| Variables^*^ | *β* (95% CI) | *P*^†^ |
| --- | --- | --- |
| ΔWMH fraction, %, per year | 0.010 (-0.007, 0.027) | 0.237 |
| ΔLacune numbers, per year | 0.090 (-0.114, 0.293) | 0.370 |
| ΔCMB counts, per year | 0.176 (-0.135, 0.487) | 0.253 |
| ΔDTI-ALPS index, per year | -0.0001 (-0.002, 0.002) | 0.903 |
| ΔPSMD, 10^-4^ mm^2^/s, per year | 0.013 (0.004, 0.023) | 0.007 |

Abbreviations: CI = confidence interval; CMB = cerebral microbleed; DTI-ALPS = diffusion tensor imaging along the perivascular space; PSMD = peak width of skeletonized mean diffusivity; WMH = white matter hyperintensity.

^*^Baseline brain age gap is an independent variable and the annual change of imaging markers including WMH fraction, lacune numbers, CMB counts, DTI-ALPS index, PSMD are dependent variables.

^†^Adjusted for age, age^2^, sex, education, and total intracranial volume in the multivariate regression analysis.

**Figure S1**


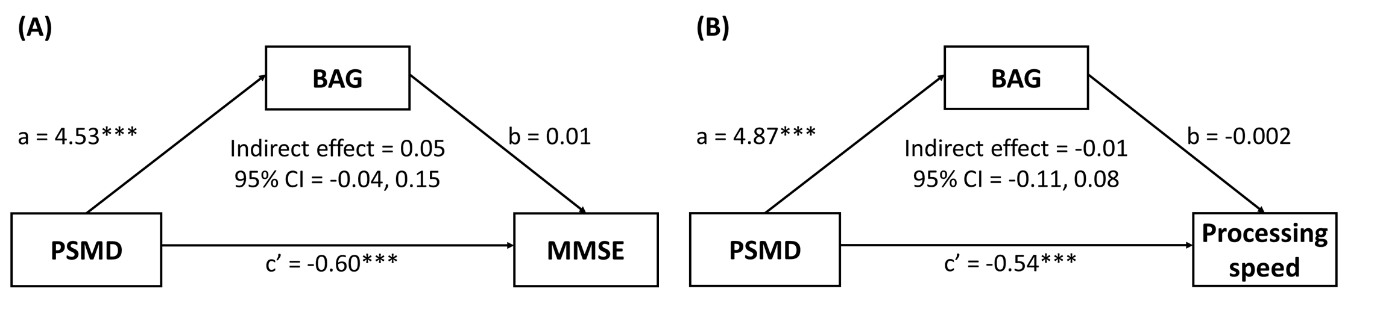


**Figure S1** Mediation models illustrating the relationship between peak width of skeletonized mean diffusivity (PSMD), brain age gap (BAG), and cognitive performance in individuals with *NOTCH3* variants. The mediation analysis was conducted using the PROCESS macro for SPSS (model 4) to evaluate the mediating effect of BAG on the relationship between PSMD and cognitive outcomes, including **(A)** Mini-Mental State Examination (MMSE) scores and **(B)** processing speed (Trail Making Test Part A). In each model, the *NOTCH3*-SVD stage serves as the independent variable, BAG acts as the mediator, and cognitive scores represent the clinical outcomes. The path coefficients a and b represent the indirect effects, while c’ represents the direct effect after adjusting for age, age², sex, education, and total intracranial volume. The indirect effect, its 95% confidence interval (CI), is provided for each model. Statistical significance was assessed using the bootstrapping method with 5,000 resamples, with mediation effects considered significant if the 95% CI did not include zero. **P* < 0.05, ***P* < 0.01, ****P* < 0.001.

**Figure S2**


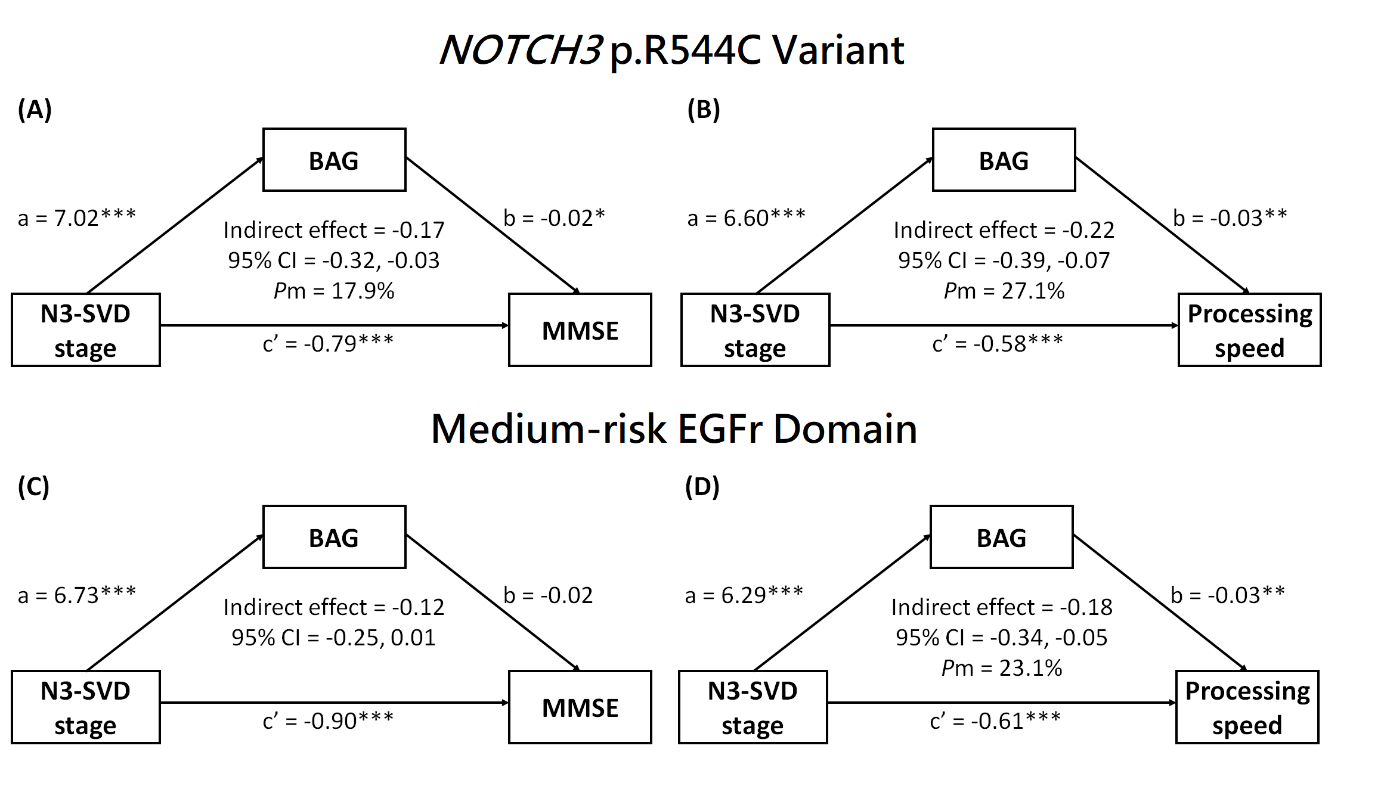


**Figure S2** Mediation models illustrating the relationship between *NOTCH3*-SVD (N3-SVD) stage, brain age gap (BAG), and cognitive performance stratified by genetic subgroups. The mediation analysis was conducted using the PROCESS macro for SPSS (model 4) to evaluate the mediating effect of BAG on the relationship between the *NOTCH3*-SVD staging system and cognitive outcomes in p.R544C carriers, including (A) Mini-Mental State Examination (MMSE) scores and (B) processing speed, and in the medium-risk EGFr domain subgroup, including (C) MMSE scores and (D) processing speed. In each model, the *NOTCH3*-SVD stage serves as the independent variable, BAG acts as the mediator, and cognitive scores represent the clinical outcomes. The path coefficients a and b represent the indirect effects, while c’ represents the direct effect after adjusting for age, age², sex, education, and total intracranial volume. The indirect effect, its 95% confidence interval (CI), and the percentage of mediation (*P*m, calculated as the ratio of the indirect effect to the total effect) are provided for each model. Statistical significance was assessed using the bootstrapping method with 5,000 resamples, with mediation effects considered significant if the 95% CI did not include zero. **P* < 0.05, ***P* < 0.01, ****P* < 0.001.


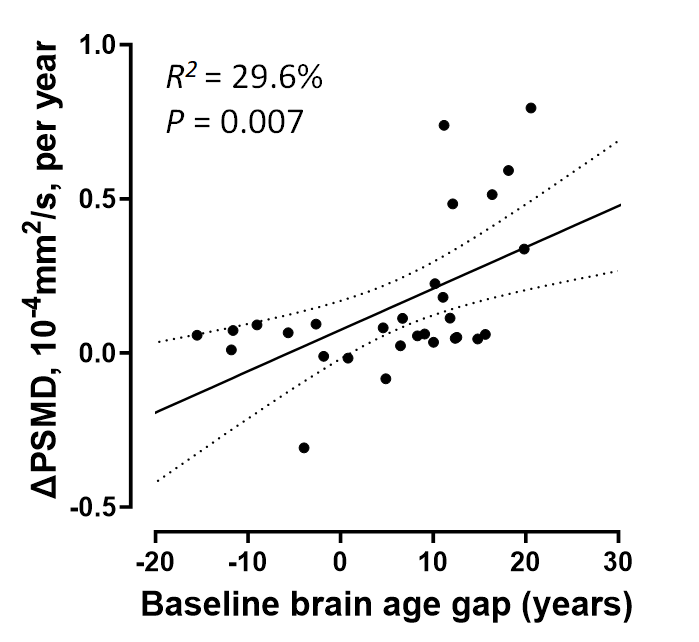


**Figure S3** Association between baseline brain age gap and the annual changes in peak width of skeletonized mean diffusivity (PSMD) in the individuals with *NOTCH3* variants. Solid lines represent linear regression fits with 95% confidence intervals (dotted lines). *R^2^* denotes the coefficient of determination; the *P* value was derived from regression analyses adjusted for age, age², sex, education, and total intracranial volume.
